# The efficacy and safety of co-administration of fimasartan and rosuvastatin to patients with hypertension and dyslipidemia

**DOI:** 10.1186/s40360-016-0112-7

**Published:** 2017-01-05

**Authors:** Moo-Yong Rhee, Taehoon Ahn, Kiyuk Chang, Shung Chull Chae, Tae-Hyun Yang, Wan Joo Shim, Tae Soo Kang, Jae-Kean Ryu, Deuk-Young Nah, Tae-Ho Park, In-Ho Chae, Seung Woo Park, Hae-Young Lee, Seung-Jea Tahk, Young Won Yoon, Chi Young Shim, Dong-Gu Shin, Hong Seog Seo, Sung Yun Lee, Doo Il Kim, Jun Kwan, Seung-Jae Joo, Myung Ho Jeong, Jin-Ok Jeong, Ki Chul Sung, Seok Yeon Kim, Sang-Hyun Kim, Kook-Jin Chun, Dong Joo Oh

**Affiliations:** 1 Cardiovascular Center, Dongguk University Ilsan Hospital, Goyang, Republic of Korea; 2 Division of Cardiology, Gachon University Gil Hospital, Incheon, Republic of Korea; 3 Division of Cardiology, Seoul St. Mary’s Hospital, Seoul, Republic of Korea; 4 Department of Internal Medicine, Kyungpook National University School of Medicine, Daegu, Republic of Korea; 5 Division of Cardiology, Inje University Busan Paik Hospital, Busan, Republic of Korea; 6 Division of Cardiology, Korea University Anam Hospital, Seoul, Republic of Korea; 7 Department of Cardiology, Dankook University Hospital, Cheonan, Republic of Korea; 8 Department of Internal Medicine, Daegu Catholic University Hospital, Daegu, Republic of Korea; 9 Division of Cardiology, Dongguk University Gyeongju Hospital, Gyeongju, Republic of Korea; 10 Department of Internal Medicine, Dong-A University College of Medicine, Busan, Republic of Korea; 11 Department of Internal Medicine, Seoul National University College of Medicine, Seoul, South Korea; 12 Division of Cardiology, Samsung Medical Center, Sungkyunkwan University School of Medicine, Seoul, Republic of Korea; 13 Division of Cardiology, Seoul National University Hospital, Seoul, Republic of Korea; 14 Department of Cardiology, Ajou University Hospital, Suwon, Republic of Korea; 15 Division of Cardiology, Gangnam Severance Hospital, Seoul, Republic of Korea; 16 Division of Cardiology, Severance Cardiovascular Hospital, Seoul, Republic of Korea; 17 Department of Cardiology, Yeungnam University Hospital, Daegu, Republic of Korea; 18 Division of Cardiology, Korea University Guro Hospital, 148, Gurodong-ro, Guro-gu, Seoul, 08308 Republic of Korea; 19 Cardiac and Vascular Center, Inje University Ilsan Paik Hospital, Goyang, Republic of Korea; 20 Department of Cardiology, Inje University Haeundae Paik Hospital, Busan, Republic of Korea; 21 Department of Cardiology, Inha University Hospital, Incheon, Republic of Korea; 22 Division of Cardiology, Jeju National University Hospital, Jeju, Republic of Korea; 23 Department of Cardiovascular, Chonnam National University Hospital, Gwangju, Republic of Korea; 24 Division of Cardiology, Chungnam National University Hospital, Daejeon, Republic of Korea; 25 Division of Cardiology, Department of Medicine, Kangbuk Samsung Hospital, Sungkyunkwan University School of Medicine, Seoul, Republic of Korea; 26 Department of Cardiology, Seoul Medical Center, Seoul, Republic of Korea; 27 Division of Cardiology, Seoul Metropolitan Government Seoul National University Hospital Boramae Medical Center, Seoul, Republic of Korea; 28 Cardiovascular Center, Pusan National University Yangsan Hospital, Yangsan, Republic of Korea

**Keywords:** Fimasartan, Rosuvastatin, Hypertension, Hypercholesterolemia

## Abstract

**Background:**

Hypertension and dyslipidemia are major risk factors of cardiovascular disease (CVD) events. The objective of this study was to evaluate the efficacy and safety of the co-administration of fimasartan and rosuvastatin in patients with hypertension and hypercholesterolemia.

**Methods:**

We conducted a randomized double-blind and parallel-group trial. Patients who met eligible criteria after 4 weeks of therapeutic life change were randomly assigned to the following groups.

1) co-administration of fimasartan 120 mg/rosuvastatin 20 mg (FMS/RSV), 2) fimasartan 120 mg (FMS) alone 3) rosuvastatin 20 mg (RSV) alone. Drugs were administered once daily for 8 weeks.

**Results:**

Of 140 randomized patients, 135 for whom efficacy data were available were analyzed. After 8 weeks of treatment, the FMS/RSV treatment group showed greater reductions in sitting systolic (siSBP) and diastolic (siDBP) blood pressures than those in the group receiving RSV alone (both *p* < 0.001). Reductions in siSBP and siDBP were not significantly different between the FMS/RSV and FMS alone groups (*p* = 0.500 and *p* = 0.734, respectively). After 8 weeks of treatment, FMS/RSV treatment showed greater efficacy in percentage reduction of low-density lipoprotein cholesterol (LDL-C) level from baseline than that shown by FMS alone treatment (*p* < 0.001). The response rates of siSBP with FMS/RSV, FMS alone, and RSV alone treatments were 65.22, 55.56, and 34.09%, respectively (FMS/RSV vs. RSV, *p* = 0.006). The LDL-C goal attainment rates with FMS/RSV, RSV alone, and FMS alone treatments were 80.43%, 81.82%, and 15.56%, respectively (FMS/RSV vs. FMS, *p* < 0.001). Incidence of adverse drug reactions with FMS/RSV treatment was 8.33%, which was similar to those associated with FMS and RSV alone treatments.

**Conclusion:**

This study demonstrated that the co-administration of fimasartan and rosuvastatin to patients with both hypertension and hypercholesterolemia was efficacious and safe.

**Trial registration:**

ClinicalTrials.gov Identifier: NCT02166814. 16 June 2014

**Electronic supplementary material:**

The online version of this article (doi:10.1186/s40360-016-0112-7) contains supplementary material, which is available to authorized users.

## Background

Hypertension and hypercholesterolemia are major risk factors of cardiovascular disease (CVD) events. The co-existence of both risk factors is quite common. The prevalence of coexistence was estimated to be 30% in an epidemiologic study [1]. The co-existence of hypertension and hypercholesterolemia can act additively or synergistically to elevate CVD risk [2, 3]. Because of the increased risk of CVD with comorbidities, guidelines have recommended simultaneous treatment of both risk factors [4, 5]. Indeed, long-term reduction of both serum total cholesterol (TC) and systolic blood pressure (SBP) by 10% could reduce major CVD events by 45% [6].

The beneficial effects for the prevention of CVD events in most clinical trials have been obtained from controlled adherence to study drugs. Poor adherence to treatment is a problem in real practice, leading to increased cardiovascular disease events [7, 8]. To improve adherence to drug treatment, regimen simplification by reducing the number of drugs and the frequency of dosing has been found to be effective. Single pill combination is one of the methods that can simplify regimens and enhance adherence to treatment [9, 10].

The present study was a phase III trial to evaluate the efficacy and safety of the co-administration of fimasartan and rosuvastatin in patients with both hypertension and hypercholesterolemia.

## Methods

### Patients

Patients (age 20–75 years) with hypertension (blood pressure ≥ 140/90 mm Hg or currently on antihypertensive medication) and dyslipidemia (defined in accordance with the National Cholesterol Education Program Adult Panel III (NCEP-ATP III) [11] or currently on lipid modifying medications) were included. Exclusion criteria were a mean sitting SBP (siSBP) ≥ 180 mmHg at screening visit and/or sitting diastolic blood pressure (siDBP) ≥ 110 mmHg; differences between arms ≥ 20 mmHg for siSBP or ≥ 10 mmHg for siDBP; secondary hypertension; secondary dyslipidemia (nephrotic syndrome, dysproteinemia, Cushing’s syndrome, and obstructive hepatopathy); fasting triglyceride level at pre-randomization visit ≥ 400 mg/dL; history of myopathy, rhabdomyolysis, and/or creatinine kinase ≥ 2× upper limit of normal; history of hypersensitivity to angiotensin receptor antagonist and/or 3-hydroxy-3-methylglutaryl coenzyme A (HMG-CoA) reductase inhibitors; gastrointestinal surgery or active inflammatory gastrointestinal diseases potentially affecting study drug absorption in the preceding 12 months; uncontrolled (glycated hemoglobin > 9% at pre-randomization visit) or insulin-dependent diabetes mellitus; liver disease (aspartate aminotransferase and/or alanine aminotransferase ≥ 2 × upper normal limit); hepatitis B (including positive test for HBsAg) or hepatitis C-positive; impaired function of kidney (serum creatinine ≥ 1.5 × upper normal limit); human immunodeficiency virus infection; electrolyte imbalance (sodium level < 133 mmol/L or ≥ 145 mmol/L or potassium level < 3.5 mmol/L or ≥ 5.5 mmol/L); retinal hemorrhage; visual disturbance or retinal microaneurysm within the past 6 months; history of abusing drugs or alcohol; ischemic heart disease within the previous 6 months (angina pectoris, acute myocardial infarction); peripheral vascular disease); percutaneous coronary intervention, or coronary artery bypass graft within the previous 6 months; severe cerebrovascular disease within previous 6 months (cerebral infarction, or cerebral hemorrhage); New York Heart Association functional class III and VI heart failure; clinically significant cardiac arrhythmia; or history of any type of malignancy within the previous 5 years; women in pregnancy, breastfeeding, or child-bearing potential without no intention of using a contraceptive.

### Study design

This multicenter, randomized, double-blind, and parallel-group trial was performed at 29 study centers in Korea. Institutional Review Board of the participating institution (Additional file 1: Table S1) and the Ministry of Food and Drug Safety approved the study design. Written informed consent was obtained from all patients. After screening, patient who met the eligible criteria entered 4 weeks of therapeutic life changes (TLC) consisting of detailed education given by a study coordinator. During the 4 weeks of TLC, patients who were already receiving lipid modifying and/or antihypertensive medications discontinued taking their lipid modifying medications for at least 4 weeks and antihypertensive medications for at least 2 weeks prior to randomization. After 4 weeks of TLC, patients who met the inclusion criteria for randomization (Table 1) were randomly assigned at a 1:1:1 ratio to receive one of three treatments once daily for 8 weeks: 1) fimasartan 120 mg/rosuvastatin 20 mg (FMS/RSV); 2) fimasartan 120 mg (FMS); 3) rosuvastatin 20 mg (RSV) using a sealed envelope with the randomization number. Study drugs were supplied by Boryung Pharmaceutical Co. Ltd. (Seoul, Republic of Korea). Randomization criteria of dyslipidemia were based on NCEP-ATP III [11].

**Table 1:** Inclusion criteria at randomization

Risk category	Cardiovascular risk factors^a^	Randomization criteria	
		LDL-cholesterol (mg/dL)	Averaged sitting SBP (mmHg)
Low risk	0 risk factors	≥160 and ≤ 250	≥140 and < 180
Moderate risk	1+ risk factors and 10 year risk < 10%	≥160 and ≤ 250	
Moderate high risk	1+ risk factors and 10-year risk from 10% to 20%	≥130 and ≤ 250	
High risk	CHD^b^ and CHD risk equivalent^c^	≥100 and ≤ 250	

^a^Risk factors: include cigarette smoking, hypertension (BP ≥ 140/90 mm Hg or on antihypertensive medication), low HDL cholesterol (<40 mg/dL), family history of premature CHD (CHD in male first-degree relative < 55 years of age; CHD in female first-degree relative < 65 years of age), and age (men ≥ 45 years; women ≥ 55 years)

^b^CHD (coronary heart disease) includes history of myocardial infarction, unstable angina, stable angina, coronary artery procedures (angioplasty or bypass surgery), or evidence of clinically significant myocardial ischemia

^c^CHD (coronary heart disease) risk equivalents include clinical manifestations of noncoronary forms of atherosclerotic disease (peripheral arterial disease, abdominal aortic aneurysm, and carotid artery disease [transient ischemic attacks or stroke of carotid origin or > 50% obstruction of a carotid artery]), diabetes, and 2+ risk factors with 10-year risk for hard CHD > 20%

All patients were instructed to orally take the assigned drug once daily in the morning for the study duration. Prior to a scheduled visit, patients were instructed to fast 12 h without taking the study drug in the morning. At each visit, three measurements of siSBP, siDBP, and pulse rate were taken from the reference arm after a 5 min rest and with a 2 min interval between measurements using a semi-automated sphygmomanometer [HEM-7080IT, Omron Corporation, Kyoto, Japan] [12, 13]. The three siSBP and siDBP measurements were averaged. Fasting blood samples obtained during scheduled visits were sent to the central laboratory (Seoul Medical Science Institute, Seoul, Korea) for analysis of TC, triglyceride, high-density lipoprotein cholesterol (HDL-C), and low density lipoprotein cholesterol (LDL-C) levels.

### Efficacy evaluation

The primary efficacy points were comparing: 1) changes from baseline in mean siSBP after 8 weeks of treatment between the FMS/RSV and RSV treatment groups, and 2) percentage change from baseline of mean LDL-cholesterol after 8 weeks of treatment between the FMS/RSV and FMS treatment groups.

The secondary efficacy points were comparing: 1) changes from baseline in mean siSBP after 8 weeks of treatment between the FMS/RSV and FMS alone treatment groups, 2) percentage change from baseline of LDL-cholesterol after 8 weeks of treatment between the FMS/RSV and RSV treatment groups, 3) changes from baseline in TC, HDL-C, and triglyceride levels after 8 weeks of treatment, 4) changes from baseline in mean siDBP after 8 weeks of treatment, 5) blood pressure control rate (the percentage of patients who reached mean siSBP < 140 mmHg after 8 weeks of treatment) and response rate (the percentage of patients who reached a mean siSBP < 140 mmHg and/or a reduction of siSBP ≥ 20 mmHg from baseline values after 8 weeks of treatment), and 6) the percentage of patients achieving the LDL-C target level after 8 weeks of treatment (goal attainment rate) according to the NCEP-ATP III guidelines (high risk: LDL-C level < 100 mg/dL; moderate/moderate high risk: LDL-C level < 130 mg/dL; low risk: LDL-C level < 160 mg/dL) [11].

### Safety evaluation

Assessment of safety and tolerability were conducted by medical examination, patient reporting, and laboratory tests (electrocardiography at baseline and the end of week 8, blood and urine tests at baseline and the end of week 8, pregnancy test at every visit). All adverse events (occurrence and elimination dates, detailed nature, duration, seriousness, intensity, significance, and relationship to the study drug) occurring during the study period were recorded.

### Sample size

This was a therapeutic confirmatory study to verify the superiority of FMS/SRV treatment in terms of change in mean siSBP (from baseline to week 8) compared to RSV alone, and in terms of percentage change of LDL-C (from baseline to week 8) compared to FMS alone. Therefore, two statistical hypotheses were formulated and the number of subjects was calculated. Total test power for the whole hypothesis was set to 80% while the two-sided significance level of each hypothesis was set to 5%. The test power for each hypothesis was set to 90% without adjusting multiplicity.

It was assumed that the mean change in siSBP with the FMS/RSV treatment was identical to that of the FMS alone treatment of a previous study, and that the mean change in siSBP of the RSV alone treatment was identical to that of the placebo. The difference in the mean and the standard deviation between the two treatment groups were estimated using weighted mean and pooled standard deviation based on the results of previous studies [14, 15]. The mean siSBP was lowered −17.41 mmHg by FMS treatment and −7.34 mmHg by placebo. The difference in siSBP lowering effect between the two groups was 10.07 mmHg with a pooled standard deviation of 12.91 mmHg. Required sample sizes were at least 36 subjects per group. A total of 135 subjects (45 subjects each for 3 groups) were considered in order to make the sample size cutoff, working under the assumption of a dropout rate of 20%.

The mean percent change of LDL-C in previous studies was −57.0% in the RSV treatment alone and −3.6% in the placebo. The difference in LDL-C lowering effect between the two groups was 53.4% with the standard deviation assumed to be 20% [16]. Required sample sizes in the comparison of the LDL-C lowering effect were at least 4 subjects per group. Therefore, a total of 15 subjects (3 groups) were required under the assumption of a dropout rate of 20%.

Finally, a total of 135 subjects were chosen as the sample size because the sample size required for the comparison of the siSBP lowering effect was greater than that required for the comparison of the LDL-C lowering effect.

### Statistical analyses

For the main analysis of efficacy, full analysis set (FAS) was used while per-protocol set (PPS) analysis was additional. FAS included all subjects with at least one efficacy evaluation result after baseline. Within the FAS, PPS consisted of patients who completed the treatment course without any significant protocol violations that might affect efficacy outcomes. If any values in the primary and secondary efficacy points were missed, the Last-Observation-Carried-Forward imputation method was used. The response rate and control rate were analyzed by using the Non-Response Imputation method to process patients with missing data at the time point of measurement as a non-responder.

The safety set included the patients who had been administered the investigational product at least once after randomization and had been assessed for safety at least once. The safety analysis was conducted based on the actual treatment group, regardless of the randomized group. Safety assessment variables for missed data were not imputed.

Using baseline values (blood pressure and LDL-C), age, gender, and smoking status as covariates, analysis of covariance (ANCOVA) was performed to test the difference in the two primary efficacy endpoints between groups at 5% significance level (two-sided). In order to test for differences within the treatment group, one sample *t*-test for the percent change in LDL-C and paired *t*-test for the change in siSBP were performed. If normality was not satisfied, a Wilcoxon signed rank test was performed.

Descriptive statistics for the secondary efficacy endpoints were presented for treatment groups. One sample *t*-test for the percentage change of LDL-C, TC, HDL-C and TG from baseline within the treatment group, and paired *t*-test for the change in siSBP and siDBP were performed. If normality was not satisfied, Wilcoxon signed rank test was performed.

The proportion and 95% confidence interval for the response rate and control rate of siSBP, as well as the goal attainment rate for LDL-C at week 8 were presented. Using the treatment groups as factors and baseline data, age, gender and smoking status as covariates, the logistic regression analysis was performed for the significant difference between monotherapy and combination therapy group.

Additionally, the interactive effect was tested by using an ANCOVA model in order to confirm the existence of an interaction effect between the monotherapy and combination treatment groups by variables. The combination therapy group was determined to be superior to the monotherapy group if both variables in the combination therapy group showed statistically significant superiority to the monotherapy group as a result of an ANCOVA test.

Medical Dictionary for Regulatory Activities (MedDRA; v 18.0) was used in coding adverse events. The percentage of patients who experienced any adverse events between groups was compared using Chi-square or Fisher's exact tests. Incidence of adverse events was presented according to severity and relationship with study drugs.

Baseline characteristics were compared among the treatment groups using Kruskal-Wallis test for continuous variables and chi-square test for categorical variables. All statistical analyses were performed using SAS® software (v 9.3; SAS Institute, Cary, NC, USA).

## Results

### Patients’ disposition

**Fig. 1:**
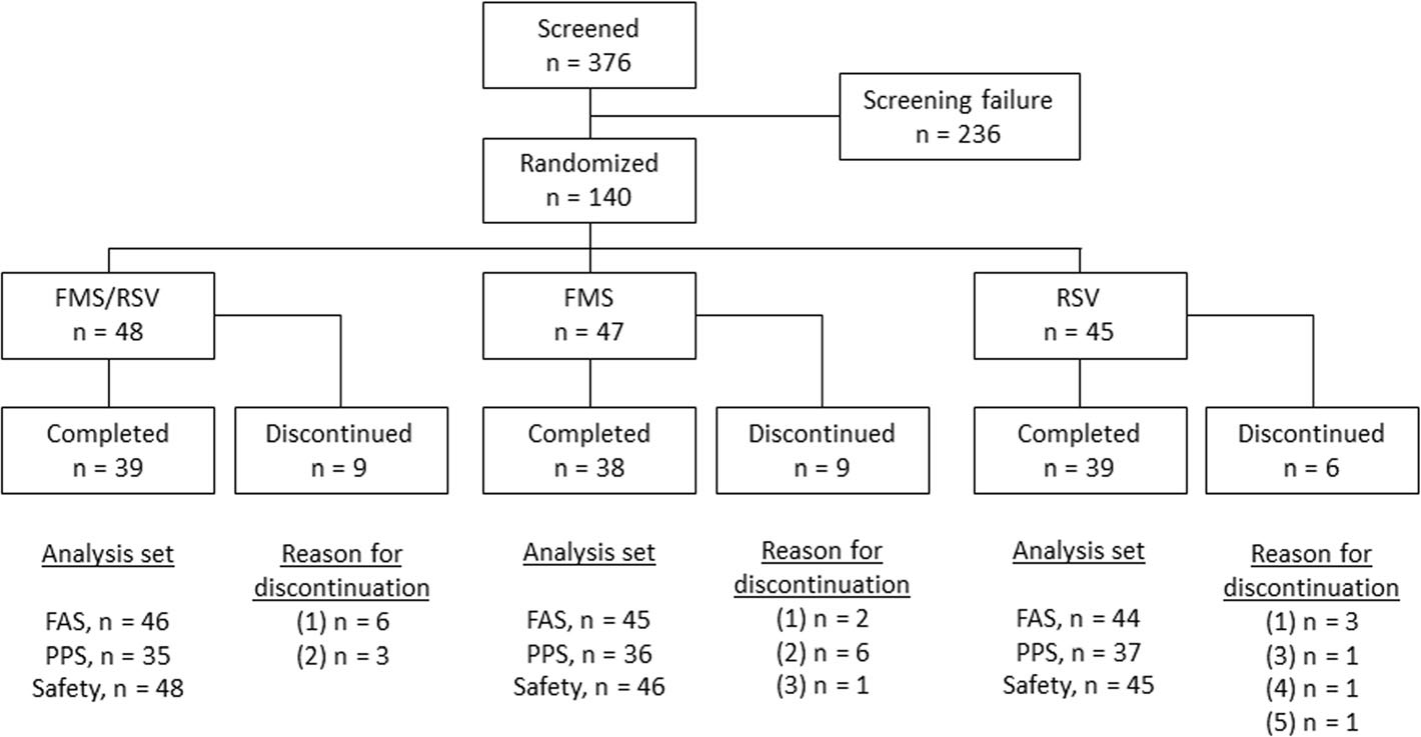
Subject disposition and reasons for study discontinuation. Reasons for discontinuation included (1) withdrawal of consent, (2) protocol violations, (3) lack of efficacy, (4) adverse events, and (5) other reasons. FMS: fimasartan; RSV, rosuvastatin. FMS/RSV: fimasartan 120 mg/rosuvastatin 20 mg treatment; FMS: fimasartan 120 mg alone treatment; RSV: rosuvastatin 20 mg alone treatment; FAS: full analysis set; PPS: per-protocol set

Among 376 screened patients, 140 were assigned to 8-week treatments of FMS/RSV, FMS alone, or RSV alone. After randomization, 24 patients discontinued the study owing to consent withdrawal (*n* = 11), protocol violation (*n* = 9), lack of efficacy (*n* = 2), adverse events (*n* = 1), and other reasons (*n* = 1) (Fig. 1). Of the 140 randomized patients, 135 were included in primary efficacy analysis after excluding five patients owing to missing efficacy data (Table 2). Their mean age was 60.5 ± 8.7 years. The majority of the study population comprised men (73.3%). The body mass index of the FMS/RSV treatment group and siDBP of the RSV alone treatment group were the highest among the three groups. Other baseline characteristics were not significantly different among the groups. Lipid-modifying agents were taken by 93 patients (68.9%). Angiotensin-converting enzyme inhibitors or angiotensin receptor blockers were taken by 81 patients (60.0%).

**Table 2:** Baseline characteristics of the study population

Demographics	Total (*n* = 135)	FMS/RSV (*n* = 46)	FMS (*n* = 45)	RSV (*n* = 44)	*P*-value
Age (year)	60.5 (8.7)	59.3 (8.7)	62.3 (9.5)	59.9 (7.7)	0.137^a^
Sex, men, n (%)	99 (73.3)	31 (67.4)	34 (75.6)	34 (77.3)	0.524^b^
Body mass index (kg/m^2^)	25.6 (2.7)	26.4 (2.8)	25.1 (2.6)	25.4 (2.8)	0.033^a^
Baseline blood pressure, mmHg					
Systolic	152.8 (9.5)	152.5 (9.9)	151.3 (9.0)	154.7 (9.5)	0.157^a^
Diastolic	89.4 (9.1)	89.4 (8.3)	85.8 (9.3)	93.1 (8.5)	0.001^a^
Baseline Pulse Rate (beats/min)	75.2 (12.1)	76.4 (13.1)	73.0 (10.9)	76.2 (12.3)	0.441^a^
Baseline LDL-C (mg/dL)	165.7 (34.6)	171.3 (36.0)	164.1 (39.6)	161.4 (26.7)	0.294^a^
Smoking, n (%)					
Current Smoker	37 (27.4)	14 (30.4)	12 (26.7)	11 (25.0)	0.933^b^
Non-smoker	57 (42.2)	17 (37.0)	2 0 (44.4)	20 (45.5)	
Ex-smoker	41 (30.4)	15 (32.6)	13 (28.9)	13 (29.6)	
Drinking, n (%)					
Current drinker	83 (61.5)	30 (65.2)	27 (60.0)	26 (59.1)	0.811^b^
Non-drinker	52 (38.5)	16 (34.8)	18 (40.0)	18 (40.9)	
Medication history of cardiovascular system, n (%)					
Lipid modifying agents	93 (68.9)	31 (67.4)	34 (75.6)	28 (63.6)	
ACE inhibitors or ARBs	81 (60.0)	28 (60.9)	29 (64.4)	24 (54.6)	
Calcium channel blockers	38 (28.2)	9 (19.6)	13 (28.9)	16 (36.4)	
Beta blockers	10 (7.4)	1 (2.2)	4 (8.9)	5 (11.4)	
Cardiac drugs	7 (5.2)	-	5 (11.1)	2 (4.6)	
Diuretics	5 (3.7)	1 (2.2)	2 (4.4)	2 (4.6)	
Peripheral vasodilators	4 (3.0)	1 (2.2)	3 (6.7)	-	
Vasoprotectives	1 (0.7)	-	-	1 (2.3)	
Medical history, n (%)					
Diabetes mellitus	33 (24.4)	8 (17.4)	16 (35.6)	9 (20.5)	
Angina pectoris	6 (4.4)	1 (2.2)	2 (4.4)	3 (6.8)	

Data are expressed as mean and standard deviation in parenthesis, and number and percent in parenthesis

*FMS/RSV* fimasartan 120 mg/rosuvastatin 20 mg treatment, *FMS* fimasartan 120 mg alone treatment, *RSV* rosuvastatin 20 mg alone treatment, *LDL-C* low-density lipoprotein cholesterol

Difference among treatment groups: ^a^Kruskal-Wallis test for continuous variables, ^b^chi-square test for categorical variables

### Efficacy

Changes in siSBP, siDBP and LDL-C are presented in Table 3. FMS/RSV combination treatment had greater efficacy in reducing siSBP from baseline after 8 weeks of treatment compared to that reported for RSV alone treatment (*p* < 0.001). Changes of siSBP was not significantly different between FMS/RSV and FMS alone groups (*p* = 0.500). Likewise, the reduction in siDBP from baseline after 8 weeks of treatment was significantly larger in the FMS/RSV treatment group compared to that in the RSV alone treatment group (*p* < 0.001). FMS/RSV and FMS alone treatments were not significantly different in the reduction of siDBP (*p* = 0.734). The least square mean (LSM) difference in siSBP and siDBP between FMS/RSV and RSV alone treatment groups was −15.03 mmHg (95% confidence interval: −21.75 to −8.31 mmHg) and −8.95 mmHg (95% confidence interval: −12.94 to −4.95 mmHg), respectively.

**Table 3:** Changes in sitting systolic blood pressure, sitting diastolic blood pressure and low-density lipoprotein cholesterol at Week 8 from baseline

	Treatment groups			FMS/RSV vs. FMS		FMS/RSV vs. RSV	
	FMS/RSV (*N* = 46)	FMS (*N* = 45)	RSV (*N* = 44)	LSM difference (SE)	*P*-value	LSM difference (SE)	*P*-value
siSBP							
Baseline	152.52 (9.85)	151.33 (8.99)	154.72 (9.46)				
Week 8	132.05 (17.45)	134.63 (18.70)	150.21 (17.19)				
Change	−20.47 (15.60)	−16.70 (16.54)	−4.50 (15.50)				
*P*-value	<0.001^a^	<0.001^b^	0.061^a^				
ANCOVA results							
LSM (SE)	−21.89 (2.44)	−19.61 (2.66)	−6.86 (2.71)	−2.28 (3.37)		−15.03 (3.39)	
(95% C.I.)	(−26.71, −17.07)	(−24.88, −14.35)	(−12.22, −1.50)	(−8.94, 4.39)	0.500^d^	(−21.75, −8.31)	<0.001^d^
siDBP							
Baseline	89.42 (8.32)	85.78 (9.30)	93.08 (8.46)				
Week 8	80.10 (10.47)	79.58 (8.47)	91.73 (11.46)				
Change	−9.32 (11.23)	−6.20 (9.98)	−1.35 (9.18)				
*P*-value	<0.001^a^	0.001^a^	0.335^a^				
ANCOVA results							
LSM (SE)	−10.13 (1.42)	−9.45 (1.59)	−1.18 (1.61)	−0.68 (1.98)		−8.95 (2.02)	
(95% C.I.)	(−12.94, −7.31)	(−12.60, −6.30)	(−4.37, 2.01)	(−4.60, 3.25)	0.734^d^	(−12.94, −4.95)	<0.001^d^
LDL-C							
Baseline	171.33 (36.02)	164.09 (39.56)	161.39 (26.71)				
Week 8	81.46 (27.10)	154.40 (52.37)	77.25 (23.33)				
Percentage change	−52.36 (12.97)	−6.52 (20.48)	−51.52 (15.80)				
*P*-value	<0.001^b^	0.112^c^	<0.001^c^				
ANCOVA results							
LSM (SE)	−52.74 (2.57)	−5.83 (2.77)	−50.92 (2.83)	−46.91 (3.53)		−1.82 (3.57)	
(95% C.I.)	(−57.82, −47.66)	(−11.31, −0.35)	(−56.52, −45.31)	(−53.89, −39.92)	<0.001^d^	(−8.89, 5.24)	0.611^d^

*FMS/RSV* fimasartan 120 mg/rosuvastatin 20 mg treatment, *FMS* fimasartan 120 mg alone treatment, *RSV* rosuvastatin 20 mg alone treatment, *LDL-C* low-density lipoprotein, *siSBP* sitting systolic blood pressure, *siDBP* sitting diastolic blood pressure, *SD* standard deviation, *LSM* least square mean, *SE* standard error, *ANCOVA* analysis of covariance

Percent change from baseline was compared by ^a^paired *t*-test, ^b^one sample *t*-test, or ^c^Wilcoxon signed rank test

^d^Comparison between the combination therapy and monotherapy was analyzed by ANCOVA model adjusted for baseline values, age, gender and smoking status

The percentage change of LDL-C from baseline to after 8 weeks of treatment was larger in the FMS/RSV treatment group than in the FMS alone treatment group (*p* < 0.001). The percentage change of LDL-C between the FMS/RSV and RSV alone treatment groups was not different (*p* = 0.611). The LSM difference of LDL-C percentage change between FMS/RSV and FMS alone treatment groups was −46.91% (95% confidence interval: −53.89 to −39.92%).

**Fig. 2:**
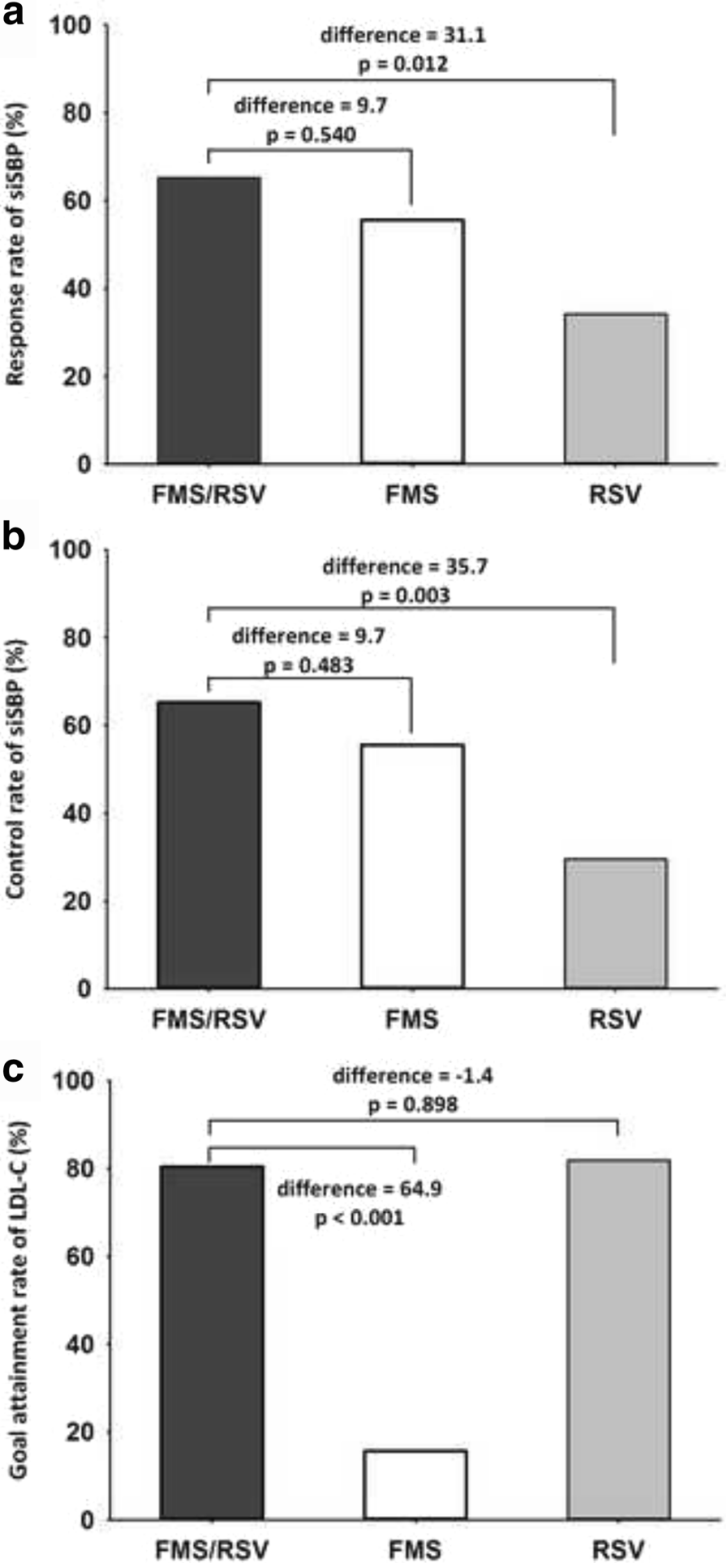
(**a**) Response rate and (**b**) control rate of sitting systolic blood pressure (siSBP), and (**c**) goal attainment rate of low-density lipoprotein cholesterol (LDL-C) by fimasartan 120 mg/rosuvastatin 20 mg (FMS/RSV) treatment, fimasartan 120 mg alone (FMS) treatment, and rosuvastatin 20 mg alone (RSV) treatment at week 8. (Full analysis set)

**Fig. 3:**
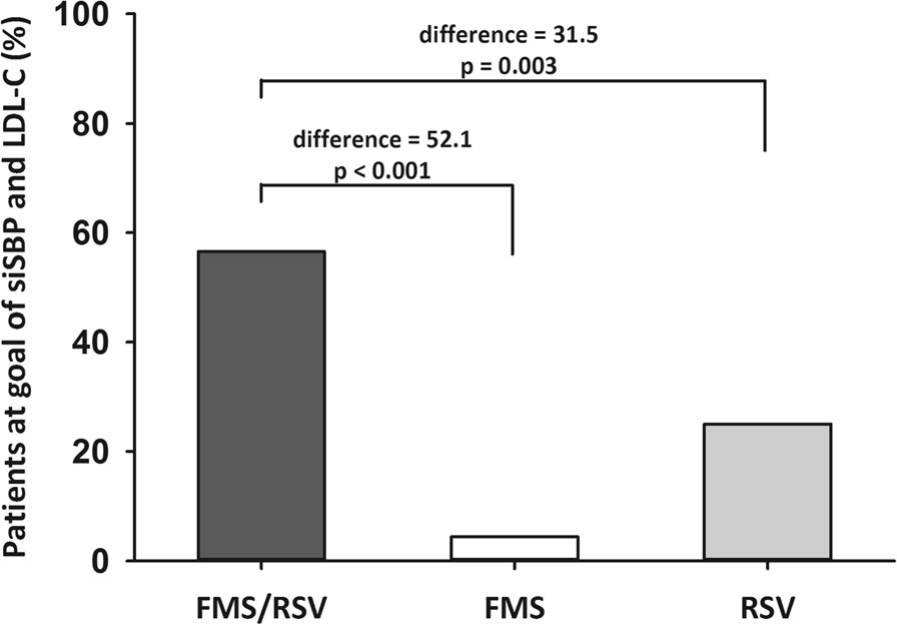
Percentage of patients who reached sitting systolic blood pressure (siSBP) and low-density lipoprotein cholesterol (LDL-C) goals after fimasartan 120 mg/rosuvastatin 20 mg (FMS/RSV) treatment, fimasartan 120 mg alone (FMS) treatment, or rosuvastatin 20 mg alone (RSV) treatment at week 8

The response rate of siSBP, control rate of siSBP, and goal attainment rate of LDL-C in FAS after 8 weeks of treatment are presented in Fig. 2. The response rate of siSBP in the FMS/RSV treatment, FMS alone treatment, and RSV alone treatment groups was 65.22, 55.56, and 34.09%, respectively (FMS/RSV vs. RSV, difference = 31.13%, 95% confidence interval 11.49 to 50.76, *p* = 0.012). The control rate of siSBP in FMS/RSV treatment, FMS alone treatment, and RSV alone treatment groups was 65.22, 55.56, and 29.55%, respectively (FMS/RSV vs. RSV, difference = 35.67%, 95% confidence interval 16.41 to 54.94, *p* = 0.003). The goal attainment rate of LDL-C in the FMS/RSV treatment, FMS alone treatment, and RSV alone treatment groups was 80.43, 15.56, and 81.82%, respectively (FMS/RSV vs. FMS, difference = 64.88%, 95% confidence interval 49.27 to 80.49, *p* < 0.001). In PPS analysis, the response and control rates of siSBP and siDBP, and the goal attainment rate of LDL-C were similar to those from FAS analysis (Table 4). The percentage of patients reaching their siSBP and LDL-C therapeutic goals after FMS/RSV treatment was 56.5%, which was higher than with FMS alone treatment (4.44%, *p* < 0.001) or with RSV alone treatment (25.0%, *p* = 0.003) (Fig. 3).

**Table 4:** Response rate and control rate of sitting systolic blood pressure, and goal attainment rate of low-density lipoprotein cholesterol by each treatment. (Analysis of per-protocol set)

	Summary of each treatment			FMS/RSV vs FMS		FMS/RSV vs RSV	
	FMS/RSV	FMS	RSV	Difference(SE)	*P*-value^d^	Difference(SE)	*P*-value^d^
	*N* = 35	*N* = 36)	*N* = 37	(95% C.I.)		(95% C.I.)	
siSBP							
Response rate^a^, n (%)	26 (74.29)	23 (63.89)	15 (40.54)	10.40 (10.89)		33.75 (10.94)	
95% CI	(59.81, 88.77)	(48.20, 79.58)	(24.72, 56.36)	(−10.95, 31.75)	0.390	(12.30, 55.19)	0.010
Control rate^b^, n (%)	26 (74.29)	23 (63.89)	13 (35.14)	10.40 (10.89)		39.15 (10.78)	
95% CI	(59.81, 88.77)	(48.20, 79.58)	(19.75, 50.52)	(−10.95, 31.75)	0.358	(18.03, 60.28)	0.003
LDL-C							
Goal attainment rate^c^, n (%)	33 (94.29)	7 (19.44)	34 (91.89)	74.84 (7.67)		2.39 (5.96)	
95% CI	(86.60, 100.00)	(6.52, 32.37)	(83.10, 100.00)	(59.80, 89.88)	<0.001	(−21.13, 25.12)	0.082

*FMS/RSV* fimasartan 120 mg/rosuvastatin 20 mg treatment, *FMS* fimasartan 120 mg alone treatment, *RSV* rosuvastatin 20 mg alone treatment, *siSBP* sitting systolic blood pressure, *LDL-C* low-density lipoprotein cholesterol, *CI* confidence interval, *SE* standard error

^a^Response Rate of siSBP: proportion of the subjects whose siSBP < 140 mmHg or Chang from Baseline of siSBP at Week 8 ≥ 20 mmHg

^b^Control rate of siSBP: proportion of the subjects whose siSBP < 140 mmHg

^c^Goal attainment rate of LDL-C: proportion of the subjects whose LDL-C < 100 mg/dL (high risk), LDL-C < 130 mg/dL (moderate high or moderate risk), LDL-C < 160 mg/dL (low risk)

^d^Comparison between combination therapy and monotherapy was analyzed by Logistic regression model adjusted for baseline values, age, and gender, smoking status

Similar to changes in LDL-C, FMS/RSV treatment also showed a greater lowering effect on TC and triglyceride, as well as HDL-C elevation compared to that reported for FMS alone treatment (Table 5).

**Table 5:** Least square mean changes in total cholesterol, high-density lipoprotein cholesterol and triglyceride from baseline after 8 weeks of treatment

	Summary of each treatment			FMS/RSV vs. FMS		FMS/RSV vs. RSV	
	FMS/RSV (*N* = 46)	FMS (*N* = 45)	RSV (*N* = 44)	LSM difference (SE)	*P*-value^a^	LSM difference (SE)	*P*-value^a^
Total cholesterol	−36.13 (1.77)	−3.62 (1.92)	−35.99 (1.95)	−32.51 (2.44)		−0.14 (2.45)	
95% CI	(−39.63, −32.64)	(−7.41, 0.17)	(−39.86, −32.13)	(−37.34, −27.69)	<0.001	(−4.99, 4.71)	0.954
HDL-C	10.24 (2.63)	−3.22 (2.86)	13.43 (2.95)	13.47 (3.63)		−3.19 (3.67)	
95% CI	(5.03, 15.45)	(−8.88, 2.43)	(7.60, 19.26)	(6.28, 20.65)	<0.001	(−10.46, 4.08)	0.387
Triglyceride	−12.78 (5.79)	20.30 (6.27)	−13.65 (6.41)	−33.08 (7.97)		0.88 (8.06)	
95%CI	(−24.24, −1.32)	(7.89, 32.71)	(−26.34, −0.96)	(−48.86, −17.30)	<0.001	(−15.08, 16.83)	0.914

*HDL-C* high-density lipoprotein cholesterol, *FMS/RSV* fimasartan 120 mg/rosuvastatin 20 mg treatment, *FMS* fimasartan 120 mg alone treatment, *RSV* rosuvastatin 20 mg alone treatment, *LSM* least square mean, *SE* standard error

^a^Comparison between the combination therapy and monotherapy was analyzed by ANCOVA model adjusted for baseline values, age, gender and smoking status

### Safety and tolerability

In the safety set (*n* = 139) analysis, the incidence of adverse events considered to be related to the study drugs was 8.63% (*n* = 12, Table 6). There was no significant difference in the incidence of study drug-related adverse events between treatment groups. Adverse events reported were dyspepsia (*n* = 1), nausea (*n* = 1), pyrexia (*n* = 1), and hepatitis (*n* = 1) in the FMS/RSV treatment group; upper abdominal pain (*n* = 1), elevation of hepatic enzyme (*n* = 1), and pollakiuria (*n* = 1) in the FMS alone treatment group; and headache (*n* = 2), hyperkalemia (*n* = 1), insomnia (*n* = 1), and pruritus (*n* = 1) in the RSV alone treatment group. There was no serious adverse event related to treatment with the study drugs.

**Table 6:** Incidence of drug related adverse events in safety analysis population

Drug related adverse events	Number (%) of subjects with ADRs			
	FMS/RSV	MFS	RSV	*p*-value
Total number (%)	4 (8.33)	3 (6.52)	5 (11.11)	0.755
Abdominal pain upper	-	1 (2.17)	-	
Dyspepsia	1 (2.08)	-	-	
Nausea	1 (2.08)	-	-	
Headache	-	-	2 (4.44)	
Pyrexia	1 (2.08)	-	-	
Hepatitis	1 (2.08)	-	-	
Hepatic enzyme increased	-	1 (2.17)	-	
Hyperkalaemia	-	-	1 (2.22)	
Insomnia	-	-	1 (2.22)	
Pollakiuria	-	1 (2.17)	-	
Pruritus	-	-	1 (2.22)	

*ADRs* adverse drug reactions, *FMS/RSV* fimasartan 120 mg/rosuvastatin 20 mg treatment, *FMS* fimasartan 120 mg alone treatment, *RSV* rosuvastatin 20 mg alone treatment

## Discussion

This study demonstrated that co-administration of FMS and RSV for 8 weeks to patients with hypertension and dyslipidemia was safe and effective in lowering blood pressure and LDL-C. The blood pressure-lowering effect of co-administration of FMS and RSV was not different from that of the FMS alone treatment, but significantly larger than that of the RSV alone treatment. The co-administration of FMS and RSV lowered LDL-C levels with similar effects as that of RSV alone treatment but significantly greater effects than that of FMS alone treatment. The response rates of siSBP and LDL-C upon co-administration of FMS and RSV were stronger than that after either RSV alone or FMS alone treatments.

Fimasartan is an antihypertensive drug that selectively blocks the angiotensin II type 1 receptor. It is used as a medication alone or in combination with other antihypertensive drugs [13, 14]. Its safety and efficacy have been proven [15, 17]. Rosuvastatin, an inhibitor of HMG-CoA reductase, can reduce LDL-cholesterol more effectively than other statins [18] with proven evidence for CVD prevention [19]. The result of this study showed that there was no interference between fimasartan and rosuvastatin on the efficacy and the safety of both drugs when they were simultaneously co-administered.

Although both antihypertensive drugs and statins have robust evidences of effect regarding the prevention of CVD, poor adherence to medications can reduce their effects in clinical practice [8, 20]. Based on data analysis of enrollees in the Korean National Health Insurance system, poor adherence to treatment in patients with hypertension has been found to be associated with increased mortality and hospitalization [7]. Among the study population, the proportion of hypertensive patients with poor (cumulative medication adherence 50 – 80%) and intermediate (cumulative medication adherence <80%) adherence to antihypertensive medications was more than 60%. The association of long-term reduction of acute CVD events with high adherence to antihypertensive treatments was revealed based on the analysis of data obtained from 400 Italian primary care physicians [20], highlighting the importance of adherence to treatment in the prevention of CVD events.

Simplification of regimens by reducing the number of drugs prescribed and the frequency of dosing is an effective method to enhance patient’s adherence to treatment [21, 22]. In a study evaluating patient adherence to hypertension medications, adherence to medication was inversely associated with the number of medications included in the regimen [23]. The levels of adherence to antihypertensive medications were 77.2%, 69.7%, 62.9%, and 55.5% in patients receiving 1-, 2-, 3-, and 4-drug regimens respectively. Single pill combination can reduce the number of medications, and has been shown to improve patient adherence to treatment by reducing pill burden [9, 10]. Single pill combination has improved compliance to medication by 21% and 26% compared to free-drug component regimen [9, 10].

A limitation of this study is that age and gender differences in blood pressure and LDL-C lowering due to different classes of antihypertensive drugs and rosuvastatin were not considered in the study design. Angiotensin converting enzyme inhibitors and beta blocker were more effective compared to calcium channel blockers and diuretics in lowering the blood pressure of young hypertensive patients (age 28–55 years) [24]. In older patients, calcium channel blockers and diuretics lowered blood pressure more than angiotensin converting enzyme inhibitors [25]. A sex difference in blood pressure control has been suggested in animal studies, but not in human studies [26]. However, effect of age and gender differences of angiotensin receptor blockers including fimasartan have not been evaluated. The effect of age and gender differences of rosuvastatin in decreasing LDL-C is controversial. A pharmacokinetic study of rosuvastatin showed a small difference in plasma concentration between age and gender groups [27]. However, this difference was not considered clinically relevant because the difference was statistically insignificant [27]. On the other hand, rosuvastatin plasma levels were significantly higher in premenopausal compared with postmenopausal women [28]. The clinical significance of different plasma levels of rosuvastatin is unclear because there is no controlled study evaluating the effect of age and gender difference of rosuvastatin treatment in lowering LDL-C. Although this study was designed without considering age and gender difference, ANCOVA model showed the age and gender independent efficacy of co-administered fimasartan and rosuvastatin.

## Conclusion

The results of this study demonstrated that co-administration of fimasartan and rosuvastatin to patients with hypertension and hypercholesterolemia was efficacious and safe. Therefore, a single pill combination of both drugs is expected to be a suitable strategy for prevention of CVD events.

## Additional file

Additional file 1: Table S1. List of Institutional Review Boards. (DOCX 18 kb)

## Abbreviations

ANCOVA: Analysis of covariance
CVD: Cardiovascular disease
FAS: Full analysis set
FMS: Fimasartan 120 mg
FMS/RSV: Fimasartan 120 mg/rosuvastatin 20 mg
HDL-C: High density lipoprotein cholesterol
HMG-CoA: 3-hydroxy-3-methylglutaryl coenzyme A
LDL-C: Low density lipoprotein cholesterol
NCEP-ATP III: National Cholesterol Education Program Adult Panel III
PPS: Per-protocol set
RSV: Rosuvastatin 20 mg
SBP: Systolic blood pressure
siDBP: Sitting diastolic blood pressure
siSBP: Sitting systolic blood pressure
TC: Total cholesterol
TLC: Therapeutic life changes
