# Supplementary material for: The efficacy and safety of co-administration of fimasartan and rosuvastatin to patients with hypertension and dyslipidemia
Source: BMC Pharmacol Toxicol. 2017 Jan 5;18:2. doi: 10.1186/s40360-016-0112-7 (PMC5217661; doi:10.1186/s40360-016-0112-7)
Supplement: Additional file 1: Table S1. — List of Institutional Review Boards. (DOCX 18 kb) [file 40360_2016_112_MOESM1_ESM.docx]

Table S1. List of Institutional Review Boards

| **Principle investigator** | **Study Centers** | **Name of Institutional Review Board** |
| --- | --- | --- |
| Moo-Yong Rhee | Dongguk University Ilsan Hospital | Dongguk University Ilsan Hospital Institutional Review Board |
| Taehoon Ahn | Gachon University Gil Medical Centerr | Gachon University Gil Hospital Institutional Review Board |
| Kiyuk Chang | Seoul St. Mary’s Hospital | Catholic Medical Center- Institutional Review Board |
| Shung Chull Chae | Kyungpook National University Hospital | Kyungpook National University Hospital Institutional Review Board |
| Tae-Hyun Yang | Inje University Busan Paik Hospital | Institutional Review Board of Inje University Busan Paik Hospital |
| Wan Joo Shim | Korea University Anam Hospital | Korea University Anam Hospital Institutional Review Board |
| Tae Soo Kang | Dankook University Hospital | Dankook University Hospital Institutional Review Board |
| Jae-Kean Ryu | Daegu Catholic University Medical Center | Daegu Catholic Medical Center Institutional Review Board |
| Deuk-Young Nah | Dongguk University Gyeongju Hospital | Dongguk University Gyeongju Hospital Institutional Review Board |
| Tae-Ho Park | Dong-A University College of Medicine | Dong-A University Hospital Institutional Review Board |
| In-Ho Chae | Seoul National University Bundang Hospital | Seoul National University Bundang Hospital Institutional Review Board |
| Seung Woo Park, | Samsung Medical Center | Samsung Medical Center Institutional Review Board |
| Hae-Young Lee | Seoul National University Hospital | Seoul National University Hospital Institutional Review Board |
| Seung-Jea Tahk | Ajou University Hospital | Ajou University Hospital Institutional Review Board |
| Young Won Yoon | Gangnam Severance Hospital | Institutional Review Board, Gangnam Severance Hospital Yonsei University College of Medicine |
| Chi Young Shim | Severance Cardiovascular Hospital | Institutional Review Board, Severance Hospital Yonsei University Health System |
| Dong-Gu Shin | Yeungnam University Hospital | Yeungnam University Hospital Institutional Review Board |
| Hong Seog Seo | Korea University Guro Hospital | Korea University Guro Hospital Institutional Review Board |
| Sung Yun Lee | Inje University Ilsan Paik Hospital | Institutional Review Board of Inje University Ilsan Paik Hospital |
| Doo Il Kim | Inje University Haeundae Paik Hospital | Institutional Review Board of Inje University Haeundae Paik Hospital |
| Jun Kwan | Inha University Hospital | Inha University Hospital Institutional Review Board |
| Seung-Jae Joo | Jeju National University Hospital | Jeju National University Hospital Institutional Review Board |
| Myung Ho Jeong | Chonnam National University Hospital | Chonnam National University Hospital Institutional Review Board |
| Jin-Ok Jeong | Chungnam National University Hospital | Chungnam National University Hospital Institutional Review Board |
| Ki Chul Sung | Kangbuk Samsung Hospital | Kangbuk Samsung Hospital Institutional Review Board |
| Seok Yeon Kim | Seoul Medical Center | Seoul Medical Center Institutional Review Board |
| Sang-Hyun Kim | Seoul Metropolitan Government, Seoul National University Boramae Medical Center | Seoul Metropolitan Government- Seoul National University Boramae Medical Center Institutional Review Board |
| Kook-Jin Chun | Pusan National University Yangsan Hospital | Pusan National University Yangsan Hospital Institutional Review Board |
| Dong Joo Oh | Korea University Guro Hospital | Korea University Guro Hospital Institutional Review Board |
